# Associations between dietary copper intake, general obesity and abdominal obesity risk: A nationwide cohort study in China

**DOI:** 10.3389/fnut.2022.1009721

**Published:** 2022-11-18

**Authors:** Weiqi Wang, Lin Liu, Ruiqi Shan, Changhong Wang

**Affiliations:** ^1^National Key Discipline, Department of Nutrition and Food Hygiene, School of Public Health, Harbin Medical University, Harbin, China; ^2^Department of Hygiene, School of Public Health, Harbin Medical University, Harbin, China; ^3^Department of Thoracic Surgery, Harbin Medical University Cancer Hospital, Harbin, China

**Keywords:** dietary copper, mineral nutrient, general obesity, abdominal obesity, cohort study

## Abstract

**Objective:**

Copper plays a crucial role in redox reactions. The aims of this research are to examine the effects of copper consumption on general obesity and abdominal obesity risk.

**Methods:**

Overall, data of 13,282 participants were obtained from the China Health and Nutrition Survey (1997–2011). A combination of individual 24-h recall and household survey was used to assess dietary intake. Time-dependent mixed effect Cox regression model treating family as a random effect were used to assess the associations between quintiles of copper intake, general obesity and abdominal obesity risk. Obesity was defined by BMI ≥ 28 kg/m2, and abdominal obesity was defined as waist circumference ≥85 cm in men and ≥80 cm in women.

**Results:**

During follow-up, 1,073 and 4,583 incident cases of general obesity and abdominal obesity occurred respectively. There were U-shaped associations of dietary copper intakes with general obesity and abdominal obesity (P for nonlinearity <0.001). In the general obesity track, compared with quintile 3 (reference category), participants in the top quintile and bottom quintile showed higher general obesity risk (HR, 2.00; 95%CI: 1.63, 2.45 for the top quintile, HR, 1.34; 95%CI: 1.08, 1.68 for the bottom quintile). In the abdominal obesity track, compared with quintile 3, the top quintile and bottom quintile were also associated with a significantly increased risk of abdominal obesity (HR, 1.68; 95%CI: 1.52, 1.87 for the top quintile, HR, 1.36; 95%CI: 1.22, 1.52 for the bottom quintile).

**Conclusions:**

We demonstrated U-shaped associations between dietary copper, general and abdominal obesity risk in Chinese and emphasized the importance of maintaining appropriate copper intake level for the prevention of obesity.

## Introduction

General obesity, as assessed by body mass index (BMI), has increased in both developed and developing countries during the past decades (1). The Global Burden of Disease (GBD) investigators showed that general obesity accounting for 4 million deaths and 120 million disability-adjusted life-years, imposing colossal burden on global health and economic system (2, 3). However, BMI alone cannot fully characterize the distribution of adiposity (4). Abdominal obesity, defined by waist circumference (WC), shows a stronger association with obesity-related complications, including type 2 diabetes, cardiovascular disease and all-cause mortality than general obesity. Although superfluous macronutrient intakes has been implicated in the etiology of obesity, growing researches suggest that dietary micronutrients like metals might also contribute to the development of obesity and related complications.

Copper, the third most abundant essential trace element in human body, mainly comes from sea food, whole cereals, fruits, and legumes (5). Numerous biochemical and crystallographic evidences have demonstrated that copper is necessary for the structural and catalytic properties of cuproenzymes, including Cu/Zn superoxide dismutase (Cu/Zn SOD), cytochrome-c oxidase (CCO), and is critical for multiple cellular pathways such as redox reaction, mitochondrial respiration and neurotransmitter synthesis (3, 6). Both insufficient and excessive copper intakes have been linked to unfavorable metabolic patterns, such as glucose intolerance, hyperlipidemia and hypertension (7, 8). Rapidly accumulating evidence suggests that copper deficiency suppresses antioxidant defense system, increases lipid peroxidation and then exacerbates detrimental effects on metabolic health (9, 10). From another aspect, as a transition metal, copper itself is an oxidant when in excess, it appears to promote oxidative stress and inflammation, which has been implicated in the pathogenesis of obesity (11). Some cross-sectional studies have evaluated the correlation between serum copper, BMI and waist, but the results were inconsistent (12). Moreover, epidemiologic evidence regarding the association between dietary copper and obesity risk has not been reported, although copper imbalance has been illustrated to affect the progression of obesity.

To address these significant gaps in knowledge outlined above, in this study, we estimate the association between dietary copper and the incidence of general obesity and abdominal obesity using longitudinal data from the China Health and Nutrition Survey (CHNS). It is hoped that our research will contribute to a better understanding of the effect of dietary copper intake on general and abdominal obesity incidence, and might have an impact on public health recommendations on copper intake for the prevention of obesity.

## Materials and methods

### Ethical approval

This study was conducted in accordance with the Declaration of Helsinki. The survey protocols, instruments, and the process for obtaining informed consent were approved by the Institutional Review Committees of the University of North Carolina at Chapel Hill, NC, USA, and the China National Institute of Nutrition and Food Safety at the Chinese Center for Disease Control and Prevention, Beijing, China. All participants provided written informed consent prior to the surveys.

### Study population

Details of the CHNS are described elsewhere (13). Briefly, the CHNS is a longitudinal household survey designed to investigate health and nutritional status in Chinese populations, and to reflect the effect of social, economic and demographic transformation of Chinese on health and nutritional status (14). The study used a multistage random-cluster sampling process to select participants from nine provinces varying according to geography, economic development and health indicators and involved eight surveys from 1991 to 2011. Each survey maintained the desired range of sociological, economic and demographic variables and new participants were recruited to replenish loss of follow-up since 1997. We analyzed CHNS data from 1997 to 2011, filtering the records in the data set based on the following criteria: age below 18 years, missing data on dietary records, pregnant women, implausible energy intake (<800 kcal/day or >5,000 kcal/day for men, <500 kcal/day or >4,000 kcal/day for women), lacking follow-up data and the extreme 1% of outliers for copper intake. After filtering, 13,282 adults remained available for the analyses. There were no appreciable numerical differences in baseline characteristics between adults in the original study population and those included in the analyses after excluding (Supplementary Table 1).

### Questionnaire survey

Detailed in-person interviews were administered by trained personnel using a structured questionnaire to collect information on demographic characteristics, dietary habits, lifestyle, physical condition and anthropometric characteristics. Dietary assessment was based on a combination of three consecutive 24 h recall at the individual level, and a food inventory taken at the household level over the same 3 day period, which could improve the accuracy of recall (15). Individuals were required to report all foods and beverages consumed both at home and away from home on a 24-h recall basis, which has been previously validated for energy intake by double-labeled water (16). At the household level, interviewers estimated the amount of each dish prepared from the household inventory and the proportion of each dish consumed by each individual, to further estimate the individual condiments intake like oil and salt. Detailed dietary data collection is described elsewhere (17). The Chinese Food Composition Table (1991) was utilized to calculate individual daily intake of select nutrients for each food item in the dietary data for surveys before 1997. A combined version of Food Composition Table (2002 and 2004) was used for the surveys from 2004 to 2011 (18). We did not include the dietary data before 1997 because the food codes in those survey years did not match the food codes in the Food Composition Table (19). Current smoking was defined as a positive answer to the question “do you still smoke cigarettes or a pipe?”, and alcohol consumption was measured using the question, “During the past year, what was your consumption (frequency and quantity) of beer, liquor and wine?” Physical activity level (PAL) was defined as the combination of occupational activity and home activity, as previously reported. The total metabolic equivalents (METs) of physical activity were calculated as MET-h per week.

### Anthropometric measurements and case ascertainment

At each survey, height was measured without shoes to the nearest 0.1 cm using a portable SECA stadiometer (SECA, Hamburg, Germany). Weight was measured without shoes and in light clothing to the nearest 0.1 kg using a calibrated beam scale. Body mass index (BMI) was calculated as weight (kg) divided by the square of the height in meters (m2). Waist was measured at a point midway between the lowest rib and the iliac crest in a horizontal plane using non-elastic tape. Waist-to-hip ratio was calculated as waist (cm) divided by hip (cm). Height, weight and waist were measured by trained examiners following a standard protocol from the World Health Organization (WHO). Height, weight and waist measurements were made at the same location and followed the same protocol at each study visit.

Obesity and abdominal obesity were defined according to the criteria recommended by Working Group on Obesity in China (WGOC) (obesity: BMI ≥ 28 kg/m^2^; abdominal obesity: waist≥ 85 cm for men and ≥ 80 cm for women) (20, 21).

### Statistical analysis

**Figure 1:**
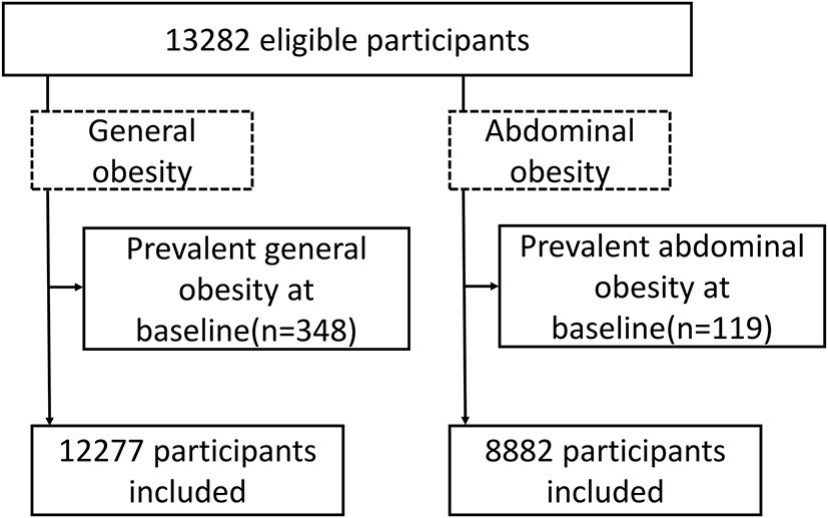
Flowchart of creating the analytical samples from CHNS, 1997–2011.

To minimize within participant variation and best reflect adult long-term diet, cumulative average copper consumption during follow-up was used in our repeated measures design study. However, we stopped updating consumption data (i.e., we used the 2000, but not the 2004 consumption information) if participants was diagnosed with obesity (i.e., in 2004 questionnaires), because participants may change their diet after development of diagnosis (22). The residual method was used to calculate the energy-adjusted dietary copper intake which was computed as the residuals from the regression model with total caloric intake as the independent variable and absolute nutrient intake as the dependent variable and then added the expected nutrient intake for the mean caloric intake of the study population. It is a desirable measure of nutrient intake independent of total caloric intake, particularly in our analysis that caloric intake is associated with obesity (23). Energy-adjusted intake was then modeled as five categorical (quintiles) variables in the main analysis. For each disease-specific analysis, prevalent cases of the corresponding disease were excluded. A flowchart is shown in Figure 1. For covariates were missing for fewer than 5% of participants, the missing values were replaced by the median values. For covariates were missing for more than 5%, the missing values were replaced by the method of multiple imputation.

The assumption of proportionality was tested by conducting correlation test between Schoenfeld residuals and survival time rank (Supplementary Table 2) (24). Moreover, log-linearity for continuous variables were examined by the Martingale residuals (Supplementary Figures 1, 2) and splines were added on variables showing non-linearity associations with obesity. On account of the within-family correlations between the family members for lifestyle factors, a mixed effect Cox regression model treating family as a random effect was performed in our study. In addition, since age changed overtime and violated the proportionality assumption in this research and, we extend to time-dependent effect model with the time^*^age interaction term in mixed effects Cox model, which was used to determine the contribution of dietary copper for the incidence of general obesity and abdominal obesity. Time at entry was age at the beginning of follow-up, and exit time was age when participants were diagnosed with cases, lost to follow-up, or censored at the end of the follow-up period, whichever came first. Models were adjusted for sex, age, smoking status, drinking status, urban or rural residence, physical activity, individual income, education level as well as energy intake. To assess the continuous association between dietary copper intake, general obesity and abdominal obesity risk and explore the shape of the dose-response relationship in a more intuitive way, we generated restricted cubic splines with 4 knots (located at the 5th, 35th, 65th, and 95th percentiles) adjusting for covariates in multivariable Cox regression. Wald test were used to detect the significance of overall association and non-linearity association in our research.

Moreover, the test of the interaction between copper intake and variables on general obesity and abdominal obesity were conducted in age (<45 vs. ≥45 years), sex, living site (urban or rural), smoking status, drinking status, magnesium intake (classified by median), zinc intake (classified by median), and iron intake (classified by median) and statistical interactions were tested by likelihood ratio test. In sensitivity analysis, considering the potential confounding caused by some metals showing similar physicochemical property with copper, we further adjusted for cumulative magnesium, zinc, and iron intake. Secondly, to minimize the potential for reverse causation, we excluded participants with events occurring during the first 2 years of follow-up. Thirdly, considering potential change of diet caused by the diagnosis of T2D and hypertension, we assessed the association between dietary copper, general obesity and abdominal obesity incidences after excluding T2D and hypertension patients. Finally, we substituted energy-adjusted copper intake with uncalibrated copper intake to see whether our findings were materially changed.

### Propensity score analysis

To address the issue of unbalanced risk factors across quintiles of cooper intake, propensity scores using variables that might affect copper intake or general obesity and abdominal obesity incidence were developed to predict the likelihood a participant would be in the different level of copper intake. We used logistic regression to calculate the probability of having lowest copper intake (quintile 1) and, in a separate analysis, of having highest copper intake (quintile 5) compared with the reference of quintile 3 since propensity score analysis requires a binary exposure variable (25). We estimated HRs using time-dependent Cox regression including the propensity score as a covariate. We also used optimize execution performance with no replacement and a caliper of 0.02 to match participants in quintile 1 (or 5, respectively) to those in quintile 3 (26). We dropped participants from our analysis without overlapping propensity scores between quintile 1 (or quintile 5, respectively) and quintile 3 and estimated HRs using time-dependent Cox regression on the exposure of being in quintile 1 (or quintile 5, respectively) compared with quintile 3 within the matched groups.

General linear models were performed to examine the differences in baseline characteristics of continuous variables across quintiles of copper consumption. χ2 test was used to measure differences in baseline characteristics of classified variables. Statistical analyses were performed using R 3.4.3 (www.r-project.org/) and SPSS 23.0 (Beijing Stats Data Mining Co. Ltd., Beijing, China). A two-sided *p*-value < 0.05 was considered statistically significant.

## Results

### Description of the study population

A total of 13,282 participants were eligible, from which were excluded prevalent cases of the disease under study. Characteristics of participants in general obesity and abdominal obesity cohort according to quintiles of cumulative copper intakes are presented in Table 1 and Supplementary Table 3. Of the 12,277 subjects in the general obesity cohort, 50.2% were women, and the mean (SD) age at baseline was 42.5 (15.4) years. Of the 8,882 subjects in the abdominal obesity cohort, 47.9% were women, and the mean (SD) age at baseline was 40.5 (15.2) years. In both cohorts, participants with higher intake of copper exhibited higher physically active levels, showed higher intake of zinc, magnesium, iron, and were more likely to be village residence.

**Table 1:** Baseline characteristics of study participants by quintiles of cumulative dietary copper intake in general obesity analysis.

**Baseline variable**	**All (*n =* 12,277)**	**Quintiles of cumulative dietary copper intake**					***P*** **of** **heterogeneity**
		**Q1 (*n =* 2,454)**	**Q2 (*n =* 2,457)**	**Q3 (*n =* 2,456)**	**Q4 (*n =* 2,455)**	**Q5 (*n =* 2,455)**	
Age (years)	42.5 (15.4)	41.8 (15.1)	42.9 (16.0)	43.3 (15.4)	42.7 (15.2)	41.7 (15.2)	<0.001
Female [(*n*, (%)]	6,166 (50.2)	1,014 (41.3)	1,242 (50.5)	1,373 (55.9)	1,284 (52.3)	1,249 (50.9)	<0.001
BMI (kg/m2)	22.2 (2.5)	22.2 (2.5)	22.0 (2.5)	22.2 (2.5)	22.1 (2.5)	22.4 (2.5)	<0.001
Waist	77.9 (8.4)	78.4 (8.3)	77.8 (8.2)	77.5 (8.4)	77.4 (8.5)	78.5 (8.5)	<0.001
WHR	0.85 (0.07)	0.86 (0.06)	0.85 (0.07)	0.85 (0.07)	0.85 (0.07)	0.85 (0.07)	<0.001
PAL (MET-h/week)	287.7 (182.0)	277.7 (185.8)	284.4 (175.8)	291.3 (177.3)	291.6 (183.1)	293.3 (187.3)	0.013
Energy intake (kcal/day)	2,267.6 (641.7)	2,382.5 (657.6)	2,203.2 (638.3)	2,192.4 (636.8)	2,221.5 (646.5)	2,338.8 (665.4)	<0.001
Cu intake (g/day)	2.1 (0.6)	1.6 (0.4)	1.9 (0.4)	2.1 (0.4)	2.3 (0.5)	2.3 (0.8)	<0.001
Mg intake (g/day)	316.4 (99.2)	272.1 (76.9)	288.4 (70.0)	312.8 (76.8)	334.3 (90.6)	374.3 (133.7)	<0.001
Zn intake (g/day)	11.8 (3.8)	11.1 (2.6)	11.7 (2.6)	11.7 (2.7)	11.8 (6.4)	12.3 (3.2)	<0.001
Fe intake (g/day)	22.8 (9.1)	20.5 (7.5)	21.9 (10.8)	23.0 (6.8)	23.5 (7.3)	25.1 (11.3)	<0.001
Living in city [(n, (%)]	4,267 (34.8)	914 (37.2)	907 (36.9)	828 (33.7)	818 (33.3)	800 (32.6)	<0.001
Urban index	57.8 (20.0)	62.7 (18.6)	59.8 (19.4)	56.8 (19.4)	55.4 (20.0)	54.3 (21.6)	<0.001
Smoking [(*n*, (%)]	3,765 (30.7)	883 (36.0)	705 (28.7)	707 (28.8)	752 (30.6)	718 (29.2)	<0.001
Drinking [(*n*, (%)]	4,251 (34.6)	944 (38.5)	833 (33.9)	796 (32.4)	822 (33.5)	856 (34.9)	<0.001
Prevalent diabetes [(*n*, (%)]	806 (6.6)	173 (7.0)	154 (6.3)	169 (6.9)	156 (6.4)	154 (6.3)	0.70
Prevalent hypertension [(*n*, (%)]	2,052 (16.7)	398 (16.2)	383 (15.6)	406 (16.5)	425 (17.3)	440 (17.9)	0.20

Continuous data are expressed as mean (SD) or number (%).

Generalized linear models and χ2 test were used to probe for differences in continuous variables and dichotomous variables.

WHR, waist-hip ratio.

### Associations of dietary copper intake with general obesity and abdominal obesity

During follow-up, we identified 1,073 incident cases of general obesity (8.7% of the general obesity cohort) and 4,583 incident cases of abdominal obesity (51.6% of the abdominal obesity cohort). In the general obesity cohort, the mean survey time was 3.80, the mean intra-person variability for copper intake was 0.51 mg/day, and the max intra-person variability was 5.83 mg/day. In the abdominal obesity cohort, the mean survey time was 3.23, the mean intra-person variability for copper intake was 0.45 mg/day, and the max intra-person variability was 4.99 mg/day.

**Figure 2:**
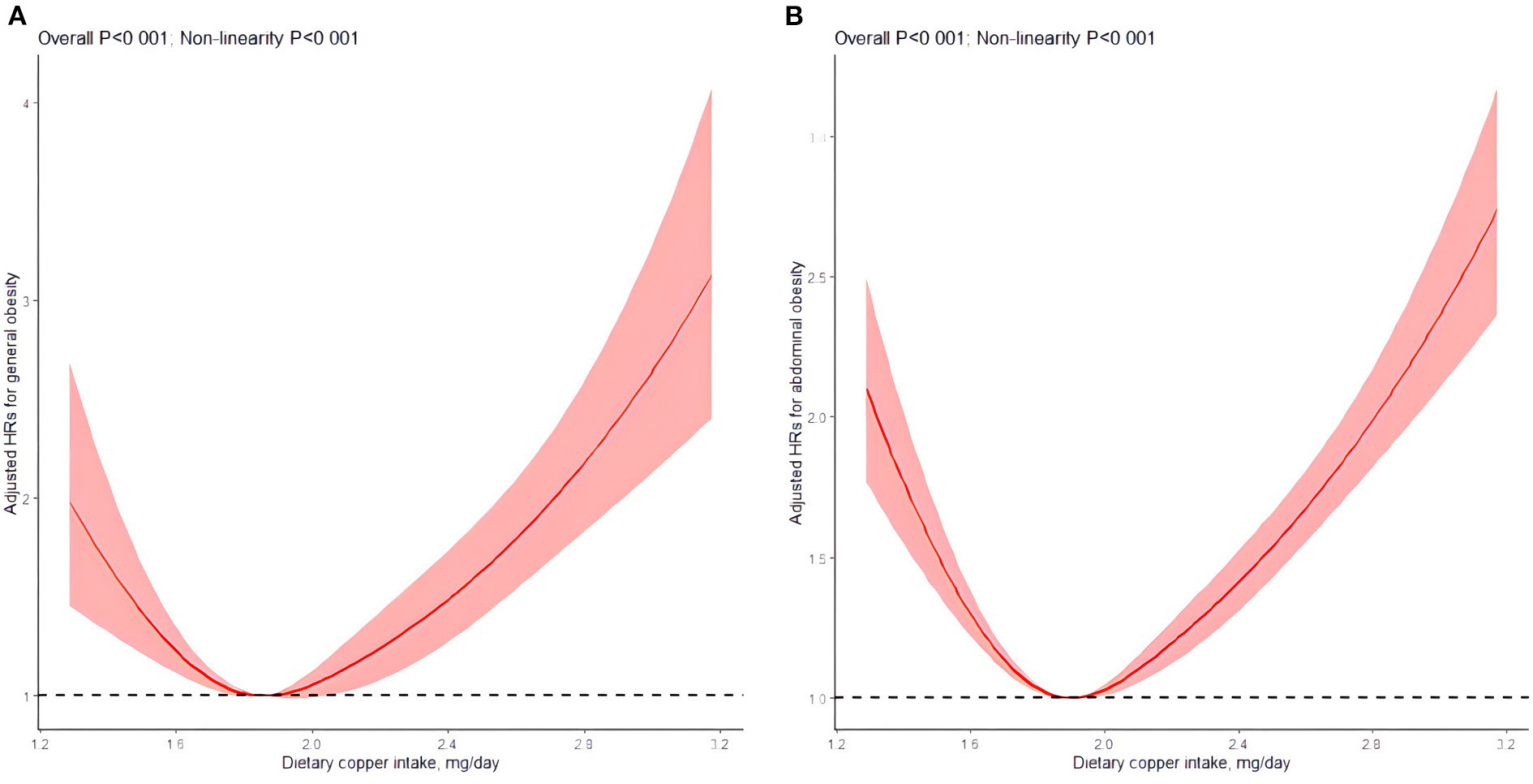
Associations of dietary copper intake with risk of general obesity **(A)** and abdominal obesity **(B)** by restricted cubic spline plots. Plots were adjusted for sex, age, smoking status, drinking status, urban or rural residence, physical activity, individual income, education level, as well as energy intake. P for overall stems from the joint Wald test for the linear and nonlinear term of RCS; P for non-linearity is derived from the Wald test for the non-linear term of the RCS.

In the general obesity track, the median follow-up duration was 8.94 (SD: 4.53) years and 7.01 (SD: 3.89) years for general obesity-free participants and general obesity participants, respectively. When dietary copper was included as a categorical variable in multivariate time-dependent Cox regression, compared with quintile 3 (1.87–2.04 mg/day), participants in quintile 1 (<1.69 mg/day) and quintile 5 (≥2.41 mg/day) showed higher general obesity risk (HR, 1.34; 95%CI: 1.08, 1.68 for quintile 1, HR, 2.00; 95%CI: 1.63, 2.45 for quintile 5) (Table 2). When dietary copper was shown in continuous form, cumulative energy-adjusted copper intake was significantly associated with general obesity in RCS (*P* <0.001). And this association followed U-shaped (P for non-linearity <0.001), such that individuals with the highest copper intake, as well as the lowest intake, had higher general obesity risk (Figure 2).

**Table 2:** Association of cumulative dietary copper intake with general obesity and abdominal obesity.

	**Quintiles of cumulative dietary copper intake**				
	**Q1**	**Q2**	**Q3**	**Q4**	**Q5**
**General obesity**					
Case [(*n*, (%)]	169 (8.0)	169 (6.9)	190 (7.7)	200 (8.1)	317 (12.9)
Univariate	1.27 (1.03–1.57)	0.94 (0.76–1.17)	1	1.06 (0.86–1.31)	2.07 (1.71–2.52)
Multivariate	1.34 (1.08–1.68)	1.07 (0.85–1.32)	1	1.06 (0.85–1.32)	2.00 (1.63–2.45)
Multivariate + Mg + Zn + Fe	1.43 (1.13–1.80)	1.09 (0.87–1.37)	1	1.04 (0.84–1.30)	1.88 (1.52–2.33)
Excluding events of first 2 years	1.36 (1.08–1.72)	1.04 (0.82–1.31)	1	1.06 (0.85–1.33)	1.97 (1.60–2.43)
Excluding participants with T2D or hypertension	1.33 (1.02–1.74)	1.03 (0.78–1.35)	1	1.12 (0.86–1.47)	1.84 (1.43–2.36)
**Abdominal obesity**					
Case [(*n*, (%)]	885 (49.8)	825 (46.5)	830 (46.7)	929 (52.3)	1,114 (62.7)
Univariate	1.37 (1.23–1.52)	1.06 (0.95–1.18)	1	1.17 (1.05–1.30)	1.85 (1.67–2.05)
Multivariate	1.36 (1.22–1.52)	1.04 (0.94–1.16)	1	1.13 (1.02–1.26)	1.68 (1.52 −1.87)
Multivariate + Mg + Zn + Fe	1.40 (1.26–1.57)	1.05 (0.95–1.17)	1	1.12 (1.01–1.24)	1.61 (1.44–1.80)
Excluding events of first 2 years	1.31 (1.17–1.46)	1.03 (0.93–1.15)	1	1.13 (1.02–1.26)	1.75 (1.57–1.94)
Excluding participants with T2D or hypertension	1.36 (1.21–1.53)	1.02 (0.91–1.15)	1	1.15 (1.02–1.29)	1.67 (1.49–1.87)

Results are presented as hazard ratio (95% confidence interval).

Time-dependent mixed effect Cox regression models including a random per-family effect were adjusted for sex, age, smoking status, drinking status, urban or rural residence, physical activity, individual income, education level as well as energy intake.

In the abdominal obesity track, the median follow-up duration was 7.91 (SD: 4.55) and 6.74 (SD: 3.70) years for abdominal obesity-free and abdominal obesity participants. In the multivariate time-dependent Cox regression, compared with quintile 3 (1.91–2.10 mg/day), participants in quintile 1 (<1.73 mg/day) and quintile 5 (≥2.37 mg/day) also showed higher abdominal obesity risk (HR, 1.36; 95%CI: 1.22, 1.52 for quintile 1, HR, 1.68; 95%CI: 1.52, 1.87 for quintile 5) (Table 2). Moreover, the association between dietary copper in continuous form and abdominal obesity was significant (*P* < 0.001), which followed U-shaped (P for non-linearity <0.001) (Figure 2).

### Stratified and sensitivity analyses

No significant interaction was detected between dietary copper intake and variables on general obesity and abdominal obesity risk. In sensitivity analysis, further adjusting for dietary magnesium, zinc and iron did not materially change the findings (Table 2). Secondly, excluding people with events during the first 2 years as well as excluding people with type 2 diabetes and (or) hypertension showed similar results with the main analysis (Table 2). Moreover, analyses using the absolute copper intake without energy-adjusted yielded consistent results (Supplementary Table 4).

### Propensity score analysis

In propensity score analysis, the researches for the associations of copper intake and general obesity and abdominal obesity were conducted in participants in quintile 1 and quintile 3 or in quintile 5 and quintile 3 of copper intake.

For general obesity, propensity score adjustment and propensity score matching did not substantially change the relationship between dietary copper and general obesity risk (Supplementary Table 5). In overall cohort, compared with quintile 3, participants in quintile 1 and quintile 5 showed higher general obesity risk (HR, 1.26; 95%CI: 1.00, 1.58 for quintile 1, HR, 1.88; 95%CI: 1.53, 2.30 for quintile 5). In the propensity-score-matched analysis that included 4,266 participants (2,133 in each group), copper intake in the lowest quintile, as compared with the middle quintile, was associated with an increased risk of general obesity (HR, 1.29; 95% CI, 1.00 to 1.65). In a similar analysis that included 4,660 subjects (2,330 in each group), participants consuming the highest quintile of copper intake showed an increased risk of general obesity (HR, 1.94; 95% CI, 1.57 to 2.40 for quintile 5), compared with the middle quintile.

For abdominal obesity, in overall cohort, compared with quintile 3, quintile 1 and quintile 5 were associated with higher risk (HR, 1.49; 95%CI: 1.33, 1.66; HR, 2.00; 95%CI: 1.79, 2.24). After propensity score matching, 3,046 (1,523 in each group) and 3,288 participants (1,644 in each group) were included, respectively. Quintile 1 and quintile 5 of copper intake were associated with higher abdominal obesity risk, as compared with quintile 3 (HR, 1.32; 95%CI: 1.17, 1.49; HR, 1.69; 95%CI: 1.51, 1.88) (Supplementary Table 5).

### Associations of dietary copper intake with BMI and waist

We further examined the associations of dietary copper intake with BMI and waist at the end of follow-up in general obesity and abdominal obesity cohort respectively. Overall, there were U-shaped associations of dietary copper with BMI and waist at the end of follow-up (*P* < 0.001 for both BMI and waist) (*P* for non-linearity = 0.003 for BMI, < 0.001 for waist) (Supplementary Figure 3). Additionally, we assessed the associations of dietary copper intake with BMI and waist change pattern. The change of BMI during follow-up showed marginally significant U-shaped association with dietary copper intake and the change of waist showed significant U-shaped association with copper (*P* < 0.001 for both BMI change and waist change) (P for non-linearity = 0.06 for BMI change, <0.001 for waist change) (Supplementary Figure 4).

## Discussion

We demonstrate U-shaped associations between dietary copper intake and risk of general obesity and abdominal obesity in a prospective, nation-wide, population-based study. The minimal general obesity and abdominal obesity risk were observed among participants with 1.87–2.04 mg/day (quintile 3) and 1.91–2.10 mg/day cooper intakes (quintile 3), respectively. Both higher and lower levels of dietary copper intakes were associated with increased risk of general obesity and abdominal obesity. Similar shapes of associations were observed for BMI and waist.

Previous studies indicated that optimal levels of copper are important for growth, metabolism, immunological and neurological function, both insufficient and excessive copper exposures are associated with unfavorable metabolic pattern (27). Our findings on the increased general obesity and abdominal obesity risk associated with low compared with moderate dietary copper intake are in line with a body of literature suggesting a causal effect of dietary copper intake on oxidative stress and lipid metabolism and with the well-established effect of oxidative stress and lipid metabolism on the development of obesity. Copper, functioning as a key cofactor of antioxidant enzymes like Cu/Zn SOD and CCO, plays a critical role in redox reactions. Copper deficiency results in the decreased activities of Cu/Zn SOD and CCO, leads to severe oxidative stress and inflammation which is induced by oxidative stress (28, 29). A research conducted in Jurkat T-lymphocytes confirmed that copper deficiency decreased cellular capability to produce Cu/Zn SOD thus increasing susceptibility to oxidative damage (30). *In vivo* study, a diet with a low copper content was found to up-regulate the activity of the pro-inflammatory protein cyclo-oxygenase-2 (31). Moreover, numerous studies have demonstrated that oxidative stress and inflammation contribute to the progress of obesity by inducing skeletal muscle dysfunction and reducing energy expenditure, as well as by inducing the resistance of leptin and insulin which could govern energy homeostasis (32, 33). No more than participated in antioxidant defense, copper is an endogenous regulator of lipolysis, which is an essential process in maintaining body weight and energy stores (34, 35). Dietary and genetic copper imbalance have been linked to misregulation of lipid metabolism in animal models. Dietary copper deficiency can lead to lipolysis activity decreasing and lipid accumulation by altering the activity of the cAMP-degrading phosphodiesterase PDE3B (36). Additionally, in rodent models, inactivation of copper-dependent enzyme semicarbazide-sensitive amine oxidase (SSAO) was reported to result in mild obesity (37, 38). In white adipose tissue, copper modulated lipolysis and governed the utilization of major metabolic fuels (fatty acids and glucose) through the activity of SSAO (36). At cellular level, limiting the availability of copper by copper chelation during adipogenesis resulted in loss of SSAO activity, unbalanced utilization of metabolic fuels, adipocyte hypertrophy and accumulation of fat (39).

We also observed an increased general and abdominal obesity risk associated with high copper intake. Plasma copper, the index of individual copper nutritional status, has been found to be positively associated with general and abdominal obesity risk in some cross-sectional studies, which provided some evidence to our results (12, 40, 41). Copper is both an antioxidant and a pro-oxidant, playing an important role as a pro-oxidant when taken in excess (7). An observational study showed close positive correlations between dietary copper, nitrotyrosine and high-sensitivity C-reactive protein levels, which are the markers of oxidative stress and inflammation (12). Besides, studies of rats reported that treatment with 60 mg/kg dietary copper could induce dyslipidemic profile and oxidative stress, which clearly emphasized the duplicitous nature of copper (11). The underlying mechanisms for the pro-oxidation capability of copper include catalyzing the formation of reactive oxygen species (ROS) *via* a Fenton-like reaction and decreasing glutathione levels (42). Furthermore, in animal models, dietary supplementation with 250 mg/kg copper increased food intake and promoted weight gain through increasing neuropeptide Y (NPY) concentration level in hypothalamus (43). Copper is an essential component for the cuproenzyme peptidylglycine monooxygenase which is responsible for the α-amidation of NPY, suggesting that the effect of copper on NPY was another likely mechanism by which excessive copper induced obesity (6). While there is no clear consensus over how excessive copper is involved in the progress of obesity, these reports raised the potential mechanism may be by enhancing oxidant stress and appetite. After comparing some food intake levels according to quintiles of dietary cumulative copper, we found that participants in Q5 showed higher fruits, whole grains, potatoes and beans intakes than in Q1, which were the main dietary source of copper. Although these are frequently regarded as apparently health foods, our study hint the importance of balanced diet for the prevention of obesity (44).

The strengths of our study included a long-term follow up with repeated dietary surveys, a relative large sample size and the prospective design. However, some limitations should be acknowledged. First, our research was conducted in Chinese, which is likely to limit the generalizability of our findings to other populations, since the range of copper intake may be disparate worldwide. Secondly, due to the observational design, residual confounding from unmeasured risk factors and reverse causality cannot be entirely ruled out. Nevertheless, adjusting multiple confounders and excluding events with 2 years in the sensitivity analyses further confirmed the robustness of our results. In addition, unlike the cumulative copper intake, the model for baseline copper intake only yielded a statistically non-significant association with obesity risk (data not shown), which might because the baseline intake showed larger measurement error for the habitual intake than cumulative intake in studies with repeated measurements (22, 45). Finally, plasma copper, markers of oxidative stress, inflammation and lipolysis were not examined in our study, which may restrict us to probe the potential mechanisms of the observed associations. Although circulating copper level has been proved to be positively associated with dietary intake and the biological mechanisms mentioned above could be plausible, we believe that it is not full and that further researches are needed to explore this point (46).

In conclusion, to our knowledge, this is the first research to explore the relationships between dietary copper, general obesity and abdominal obesity. We demonstrate a U-shaped association between dietary copper intake, general obesity and abdominal obesity risk in this large prospective cohort study. Similar shapes of associations are discovered for BMI and waist. Our research emphasizes the importance of maintaining the optimal copper intake level to prevent obesity risk and may provide valuable information for public health recommendations on copper intake. Further studies are needed to apply these findings widely in public health practice.

## Data availability statement

The original contributions presented in the study are included in the article/Supplementary material, further inquiries can be directed to the corresponding author.

## Ethics statement

The study was approved by the Institutional Review Committees of the University of North Carolina at Chapel Hill, and the China National Institute of Nutrition and Food Safety at the Chinese Center for Disease Control and Prevention. The patients/participants provided their written informed consent to participate in this study.

## Author contributions

WW and RS conceived the experiment. WW and LL contributed to the data analysis. WW drafted the manuscript. CW reviewed the manuscript. All authors were involved in writing the paper and had final approval of the submitted version.

## Conflict of interest

The authors declare that the research was conducted in the absence of any commercial or financial relationships that could be construed as a potential conflict of interest.

## Publisher's note

All claims expressed in this article are solely those of the authors and do not necessarily represent those of their affiliated organizations, or those of the publisher, the editors and the reviewers. Any product that may be evaluated in this article, or claim that may be made by its manufacturer, is not guaranteed or endorsed by the publisher.
