# Supplementary material for: Associations between dietary copper intake, general obesity and abdominal obesity risk: A nationwide cohort study in China
Source: Front Nutr. 2022 Nov 18;9:1009721. doi: 10.3389/fnut.2022.1009721 (PMC9716269; doi:10.3389/fnut.2022.1009721)
Supplement: Supplementary file 1 [file Data_Sheet_1.docx]

Supplementary Material

# Supplementary Figures


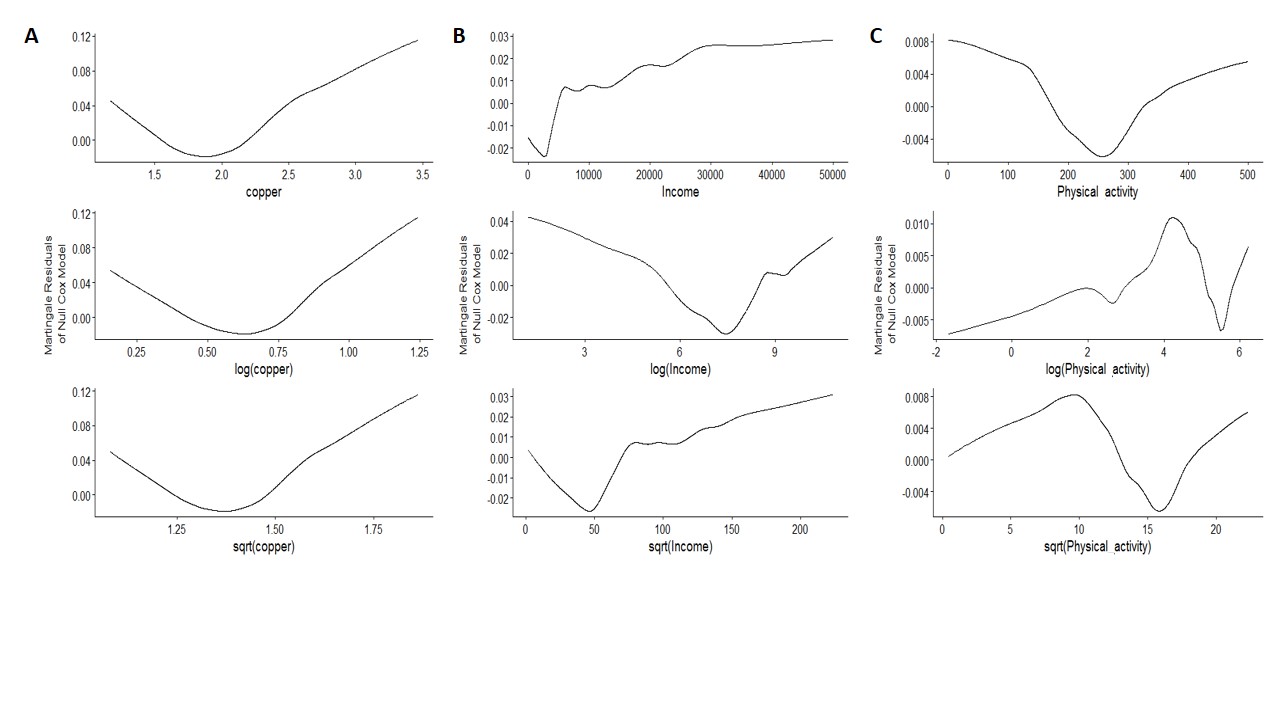
**Supplementary Figure 1.** Associations of dietary copper intake (A), individual income (B), and Physical activity (C) with general obesity risk by Martingale residuals plots.


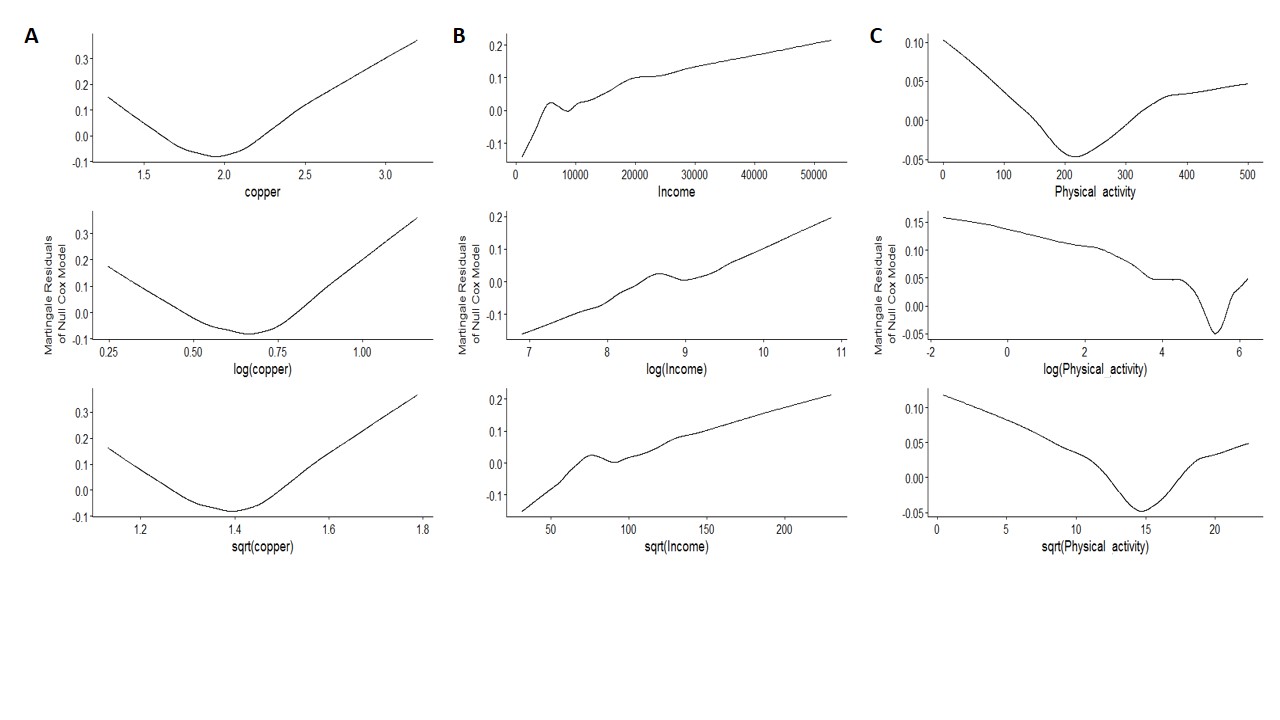
**Supplementary Figure 2.** Associations of dietary copper intake (A), individual income (B), and Physical activity (C) with abdominal obesity risk by Martingale residuals plots.


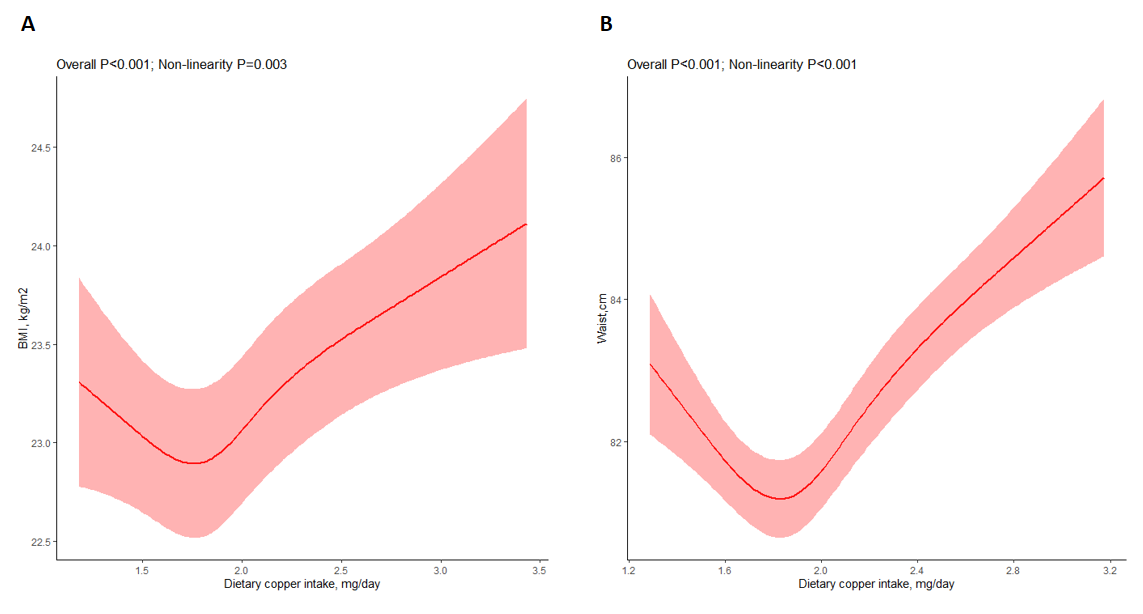


**Supplementary Figure 3.** Associations of dietary copper intake with BMI **(A)** and waist **(B)** at the end of follow-up by restricted cubic spline plots. Plots were adjusted for sex, age, smoking status, drinking status, urban or rural residence, physical activity, individual income, education level as well as energy intake.


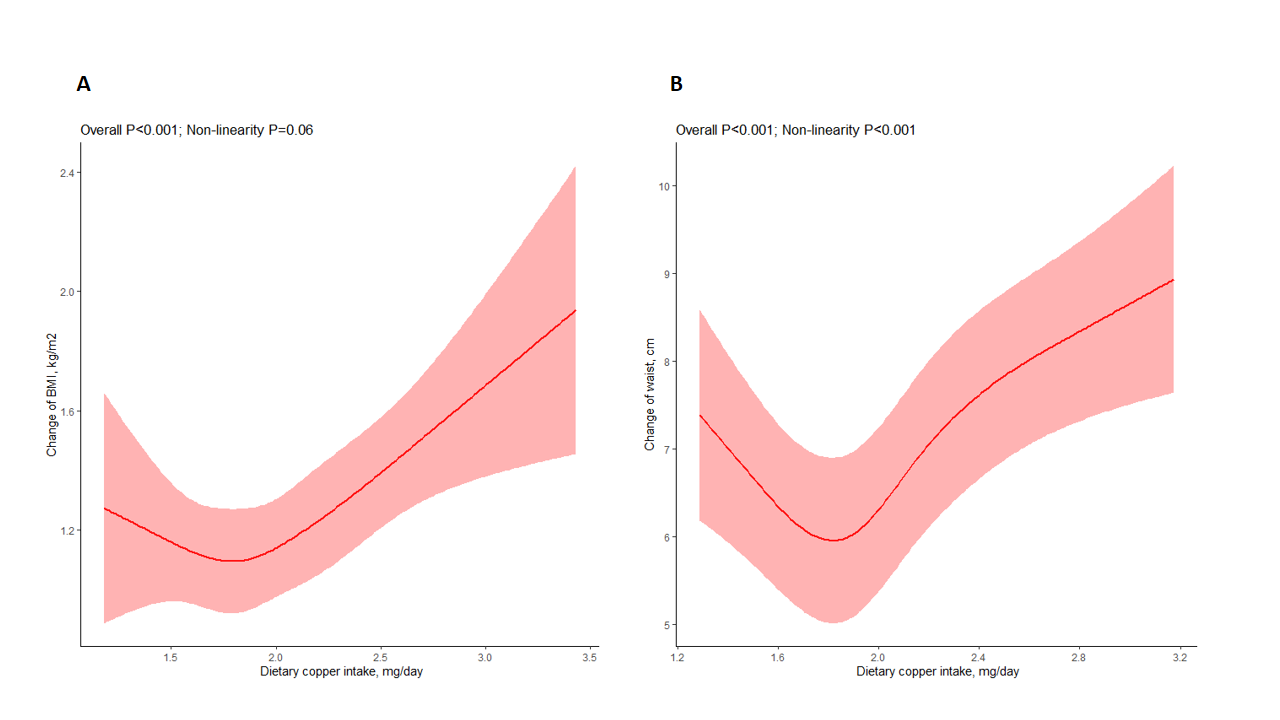


**Supplementary Figure 4.** Associations of dietary copper intake with the change of BMI **(A)** and waist **(B)** during follow-up by restricted cubic spline plots. Plots were adjusted for sex, age, smoking status, drinking status, urban or rural residence, physical activity, individual income, education level as well as energy intake.

# Supplementary Tables

Supplementary Table 1 Baseline characteristics of original adult population and study population

| **Baseline variable** | Original adult population(n=20747) | Study population(n=13282) | P value |
| --- | --- | --- | --- |
| Age (years) | 43.6(15.8) | 42.8(15.4) | <0.001 |
| Female [(n, (%)] | 11035(53.2) | 6663(50.2) | <0.001 |
| BMI (kg/m2) | 23.0(3.6) | 22.6(3.2) | <0.001 |
| Waist | 80.2(10.7) | 78.9(9.9) | <0.001 |
| WHR | 0.86(0.09) | 0.85(0.07) | <0.001 |
| PAL(MET-h /week) | 297.0(218.6) | 286.9(181.7) | <0.001 |
| Energy intake (kcal/day) | 2186.3(1185.8) | 2275.9(657.9) | <0.001 |
| Cu intake (g/day) | 2.0(1.4) | 2.1(0.9) | <0.001 |
| Protein intake (g/day) | 67.5(29.0) | 68.7(23.1) | <0.001 |
| Fat intake (g/day) | 72.9(110.8) | 68.8(36.4) | <0.001 |
| Carbohydrate intake (g/day) | 316.9(135.4) | 344.7(121.4) | <0.001 |
| High school education [(n, (%)] | 3115(15.6) | 1312(10.1) | <0.001 |
| Urban index | 63.5(21.3) | 58.4(20.1) | <0.001 |
| Drinking [(n, (%)] | 7066(34.1) | 4631(34.9) | 0.1 |
| Prevalent diabetes [(n, (%)] | 1292(6.2) | 989(7.4) | <0.001 |
| Prevalent hypertension [(n, (%)] | 4244(20.5) | 2465(18.6) | <0.001 |

Continuous data are expressed as mean (SD) or number (%)

Generalized linear models and χ2 test were used to probe for differences in continuous variables and dichotomous variables

WHR: waist-hip ratio

Supplementary Table 2 The test of proportional assumption for variables in general obesity cohort and abdominal obesity cohort.

| Variables | P value in general obesity cohort | P value in abdominal obesity cohort |
| --- | --- | --- |
| Age (years) | 0.3 | 0.7 |
| Female [(n, (%)] | <0.001 | <0.001 |
| Smoking [(n, (%)] | 0.7 | 0.8 |
| Drinking [(n, (%)] | 0.3 | 0.4 |
| Live in urban [(n, (%)] | 0.3 | 0.6 |
| PAL(MET-h /week) | 0.4 | 0.3 |
| Individual income (yuan) | 0.5 | 0.5 |
| High school education [(n, (%)] | 0.3 | 0.3 |
| Energy intake (kcal/day) | 0.2 | 0.05 |

Supplementary Table 3 Baseline characteristics of study participants by quintiles of cumulative dietary copper intake in abdominal obesity analysis

| **Baseline variable** | All(n=8882) | Quintiles of cumulative dietary copper intake | | | | | P of heterogeneity |
| --- | --- | --- | --- | --- | --- | --- | --- |
|  |  | Q1(n=1777) | Q2(n=1776) | Q3(n=1776) | Q4(n=1777) | Q5(n=1776) |  |
| Age (years) | 40.5(15.2) | 40.0(15.0) | 40.4(15.9) | 41.0(15.3) | 41.1(15.1) | 39.8(14.7) | 0.036 |
| Female [(n, (%)] | 4235(47.9) | 700(39.4) | 871(49.0) | 913(51.4) | 881(49.6) | 888(50.0) | <0.001 |
| BMI (kg/m2) | 21.4(2.2) | 21.4(2.1) | 21.2(2.2) | 21.2(2.1) | 21.3(2.2) | 21.7(2.1) | <0.001 |
| Waist | 74.0(5.6) | 74.5(5.7) | 73.9(5.4) | 73.5(5.7) | 73.7(5.7) | 74.6(5.4) | <0.001 |
| WHR | 0.83(0.05) | 0.84(0.05) | 0.83(0.05) | 0.83(0.05) | 0.83(0.05) | 0.83(0.05) | <0.001 |
| PAL(MET-h /week) | 285.8(181.2) | 275.5(187.1) | 288.6(290.9) | 290.9(176.4) | 281.2(173.1) | 292.8(187.6) | 0.02 |
| Energy intake (kcal/day) | 2285.0(649.6) | 2428.2(627.2) | 2201.0(617.1) | 2192.6(646.5) | 2203.7(633.0) | 2399.6(679.6) | <0.001 |
| Cu intake (g/day) | 2.1(0.6) | 1.6(0.3) | 1.9(0.3) | 2.1 (0.4) | 2.3(0.4) | 2.8(0.7) | <0.001 |
| Mg intake (g/day) | 319.1(95.4) | 267.0(75.1) | 287.9(67.1) | 312.0(73.7) | 337.8(83.6) | 388.0(120.0) | <0.001 |
| Zn intake (g/day) | 11.9(2.9) | 11.4(3.2) | 11.9(2.4) | 11.8(2.2) | 11.9(3.4) | 12.5(3.0) | <0.001 |
| Fe intake (g/day) | 22.8(8.8) | 20.3(7.2) | 21.9(11.8) | 22.9(6.6) | 23.3(6.6) | 25.8(9.5) | <0.001 |
| Living in city [(n, (%)] | 2838(32) | 645(36.3) | 627(35.3) | 548(30.9) | 532(29.9) | 486(27.4) | <0.001 |
| Urban index | 55.5(19.7) | 61.2(18.6) | 58.2(19.2) | 55.3(19.1) | 52.6(19.1) | 50.4(20.4) | <0.001 |
| Smoking [(n, (%)] | 2855(32.1) | 648(36.5) | 527(29.7) | 557(31.4) | 564(31.7) | 559(31.5) | <0.001 |
| Drinking [(n, (%)] | 3118(25.1) | 663(37.3) | 618(34.8) | 592(33.3) | 611(34.4) | 634(35.7) | 0.14 |
| Prevalent diabetes [(n, (%)] | 454(5.1) | 95(5.3) | 85(4.8) | 87(4.9) | 92(5.2) | 95(5.3) | 0.91 |
| Prevalent hypertension [(n, (%)] | 1039(11.7) | 195(11.1) | 198(11.1) | 213(12.0) | 207(11.6) | 226(12.7) | 0.50 |

Continuous data are expressed as mean (SD) or number (%)

Generalized linear models and χ2 test were used to probe for differences in continuous variables and dichotomous variables

WHR: waist-hip ratio

Supplementary Table 4 Association between uncalibrated cumulative dietary copper intake, general obesity and abdominal obesity

|  | Quintiles of uncalibrated cumulative dietary copper intake | | | | |
| --- | --- | --- | --- | --- | --- |
|  | Q1 | Q2 | Q3 | Q4 | Q5 |
| **General obesity** |  |  |  |  |  |
| Case [(n, (%)] | 190(7.7) | 181(7.4) | 160(6.5) | 237(9.6) | 305(12.4) |
| HR | 1.47(1.15-1.86) | 1.20(0.95-1.51) | 1 | 1.48(1.19-1.85) | 2.28 (1.83-2.84) |
| **Abdominal obesity** |  |  |  |  |  |
| Case [(n, (%)] | 893(50.3) | 833(46.9) | 837(47.2) | 932(52.4) | 1088(61.3) |
| HR | 1.34(1.20-1.50) | 0.92(0.83-1.02) | 1 | 1.13(1.02-1.26) | 1.70(1.53-1.89) |

Results are presented as hazard ratio (95% confidence interval).

Models were adjusted for sex, age, smoking status, drinking status, urban or rural residence, physical activity, individual income, education level as well as energy intake.

Supplementary Table 5 Association between cumulative dietary copper intake, general obesity and abdominal obesity, using propensity score analyses

|  | Quintile 1 vs 3 | | Quintile 5 vs 3 | |
| --- | --- | --- | --- | --- |
|  | Case [(n, (%)] | HR(95%CI) | Case [(n, (%)] | HR(95%CI) |
| **General obesity** |  |  |  |  |
| Overall cohort | 197(8.0) vs 190(7.7) |  | 317(12.9) vs 190(7.7) |  |
| Multivariate |  | 1.35(1.06-1.70) |  | 1.97(1.60-2.43) |
| Propensity score adjusted |  | 1.26(1.00-1.58) |  | 1.88(1.53-2.30) |
| Matched by propensity score | 178(8.3) vs162(7.6) | 1.29(1.00-1.65) | 305(12.8) vs 188(7.9) | 1.94(1.57-2.40) |
| **Abdominal obesity** |  |  |  |  |
| Overall cohort | 885(49.8) vs 830(46.7) |  | 1117(62.7) vs 830(467) |  |
| Multivariate |  | 1.34(1.19-1.50) |  | 1.72(1.54-1.94) |
| Propensity score adjusted |  | 1.49(1.33-1.66) |  | 2.00 (1.79-2.24) |
| Matched by propensity score | 759(49.8) vs 718(47.1) | 1.32(1.17-1.49) | 1030(62.7) vs 787(47.9) | 1.69(1.51-1.88) |

Multivariable analysis were adjusted for sex, age, smoking status, drinking status, urban or rural residence, physical activity, individual income, education level as well as energy intake.

Propensity scores are based on the covariates included in multivariate Cox model except age.
